# Tropical Indo-Pacific SST influences on vegetation variability in eastern Africa

**DOI:** 10.1038/s41598-021-89824-x

**Published:** 2021-05-17

**Authors:** In-Won Kim, Malte F. Stuecker, Axel Timmermann, Elke Zeller, Jong-Seong Kug, So-Won Park, Jin-Soo Kim

**Affiliations:** 1 Center for Climate Physics, Institute for Basic Science, Busan, Republic of Korea; 2 Pusan National University, Busan, Republic of Korea; 3 Department of Oceanography and International Pacific Research Center, School of Ocean and Earth Science and Technology, University of Hawaiʻi at Mānoa, Honolulu, HI USA; 4 Division of Environmental Science and Engineering, Pohang University of Science and Technology, Pohang, Republic of Korea; 5 Department of Evolutionary Biology and Environmental Studies, University of Zurich, Zurich, Switzerland

**Keywords:** Atmospheric science, Biogeochemistry, Hydrology

## Abstract

Mechanisms by which tropical Pacific and Indian Ocean sea surface temperatures (SST) influence vegetation in eastern Africa have not been fully explored. Here, we use a suite of idealized Earth system model simulations to elucidate the governing processes for eastern African interannual vegetation changes. Our analysis focuses on Tanzania. In the absence of ENSO-induced sea surface temperature anomalies in the Tropical Indian Ocean (TIO), El Niño causes during its peak phase negative precipitation anomalies over Tanzania due to a weakening of the tropical-wide Walker circulation and anomalous descending motion over the Indian Ocean and southeastern Africa. Resulting drought conditions increase the occurrence of wildfires, which leads to a marked decrease in vegetation cover. Subsequent wetter La Niña conditions in boreal winter reverse the phase in vegetation anomalies, causing a gradual 1-year-long recovery phase. The 2-year-long vegetation decline in Tanzania during an ENSO cycle can be explained as a double-integration of the local rainfall anomalies, which originate from the seasonally-modulated ENSO Pacific-SST forcing (Combination mode). In the presence of interannual TIO SST forcing, the southeast African precipitation and vegetation responses to ENSO are muted due to Indian Ocean warming and the resulting anomalous upward motion in the atmosphere.

## Introduction

Natural fluctuations in Africa’s vegetation are affected by rainfall variability^1–7^. Especially the Sahel, eastern Africa, and southern Africa show large interannual variations in terrestrial productivity, which can be attributed to year-to-year changes in water stress^8^. Linking African terrestrial rainfall variability to large-scale climate variability might result in potential predictability of associated vegetation changes. Especially the El Niño-Southern Oscillation (ENSO) is an important climate driver of interannual rainfall variability in parts of Africa. On average, El Niño events cause droughts in southern Africa and enhanced precipitation and corresponding floods in eastern Africa^9^. An associated vegetation response can be identified by the relationship between ENSO and the Normalized Difference Vegetation Index (NDVI) over both these regions^10–14^. In contrast, over the Sahel, the relationship between ENSO and NDVI is weak^15^. Interannual vegetation changes over eastern Africa show a nonlinear relationship with rainfall variability and a strong dependency on land cover type is observed^2^. Globally, an asymmetric relationship between net primary production and rainfall is observed for grasslands^16^. These examples highlight that the vegetation response to climate factors is often modulated by nonlinear land processes.

Wildfires can play an important role in these interannual vegetation changes through climate-fire-vegetation interactions^17,18^. For wet savannas in Africa, an increase in rainfall-induced fuel moisture leads to a decrease in the burned area^7^, while for dry savannas, an increase in moisture promotes biomass as a role of fuels, thereby facilitating more fires^19^. The roles of climate and fire in African vegetation dynamics were further highlighted in a series of fire-off experiments^20,21^, and the vegetation model experiments with fire modules realistically represented vegetation compared to the fire-off experiments.

In addition to potential nonlinear effects caused by vegetation types and fire activity mentioned above, observations and model experiments reveal a spatial asymmetry in atmospheric response over Africa between El Niño and La Niña^22^. Furthermore, El Niño and La Niña events also influence the pattern and seasonal evolution of NDVI in eastern Africa in an asymmetric way^12^. In addition, nonlinear ENSO teleconnections to Africa might further be affected by ENSO-induced asymmetric sea surface temperature (SST) responses over the Atlantic and Indian Oceans. For instance, SST variability over the southern Atlantic and tropical Pacific Ocean have a negative relationship with rainfall over the Sahel^10^. Indian Ocean Dipole (IOD) events, typically accompanied by ENSO^23^, positively correlate with eastern African rainfall during the short rainy season^24,25^.

Although the aforementioned studies have demonstrated the impacts of ENSO on African vegetation based on observations, we still lack a deeper understanding on the involved physical processes, including the role of wildfires. Moreover, the direct ENSO impacts (i.e., caused by SST anomalies in the tropical Pacific Ocean) are difficult to delineate from indirect ENSO impacts (i.e., caused by ENSO-induced SST anomalies in the Indian Ocean) based on observations alone. Therefore, in this study, we investigate the vegetation response in sub-Saharan Africa to both direct and indirect ENSO forcing through a series of targeted model experiments and compare them to the observations.

## Results

### The Walker circulation response to ENSO

**Figure. 1:**
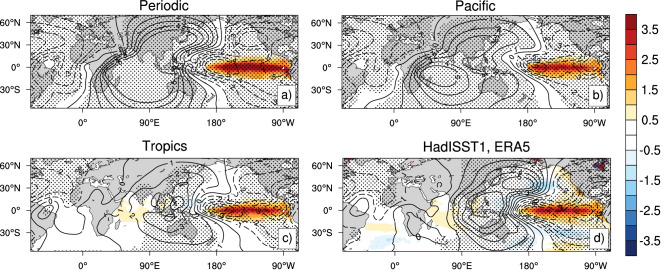
Composite differences of SST anomalies (shading; unit: K), and 200 hPa velocity potential anomalies in D(0)JF(1) (contours; unit: m^2^ s^−1^, scaled by 10^6^) between El Niño and La Niña events for the Periodic experiment (**a**), the Pacific experiment (**b**), the Tropics experiment (**c**), HadISST1 and ERA5 reanalysis (**d**): El Niño events: 1982, 1987, 1991, 1997, 2002, 2009; La Niña events: 1984, 1988, 1999, 2000, 2007, 2010 for the Pacific experiment, the Tropics experiment, and the observations. Negative values (dashed lines) indicate anomalous upward motion and positive values (solid lines) indicate anomalous downward motion. Stippling indicates a statistically significant difference at the 95% significance level for the 200 hPa velocity potential. Fig. 1 was generated using NCAR Command Language Version 6.5.0 (http://dx.doi.org/10.5065/D6WD3XH5).

To investigate the drivers of the vegetation response over Africa to Indo-Pacific SST anomalies, we first focus on the tropical large-scale atmospheric circulation and its interannual variations. The position and strength of the Walker circulation are closely coupled to SST anomalies in the tropical Pacific. Both the “Periodic” and the “Pacific” experiments (SST anomalies are only prescribed in the tropical Pacific) show pronounced Walker circulation changes between El Niño and La Niña events with anomalous ascending motion over the eastern Pacific region (and corresponding upper-level divergence) and anomalous descending motion (and corresponding upper-level convergence) during the peak ENSO phase of December–January–February [D(0)JF(1)] (Fig. 1a,b). Importantly, the edge of the descending motion extends to the African continent in the two experiments. In contrast, the “Tropics” experiment shows that the center of the descending motion shifts toward the Maritime Continent, inducing weaker subsidence around the Indian and Atlantic Ocean, accompanying tropical Indian Ocean (TIO) warming (Fig. 1c). This large-scale circulation response is similar to what is seen for the observations (Fig. 1d). The TIO warming pattern seen in Fig. 1c,d is largely forced by El Niño and then is prolonged for several months after the El Niño event due to the so-called capacitor effect^26,27^. The pattern of large-scale atmospheric anomalies in the Tropics experiment (Fig. 1c) is more consistent with the observations (Fig. 1d) than the Periodic and Pacific experiments (Fig. 1a, b). This suggests that TIO warming affects the change of the large-scale atmospheric circulation around the African continent related to ENSO, as suggested previously^28^.

### The response of rainfall, vegetation, and wildfire over Africa to ENSO

**Figure. 2:**
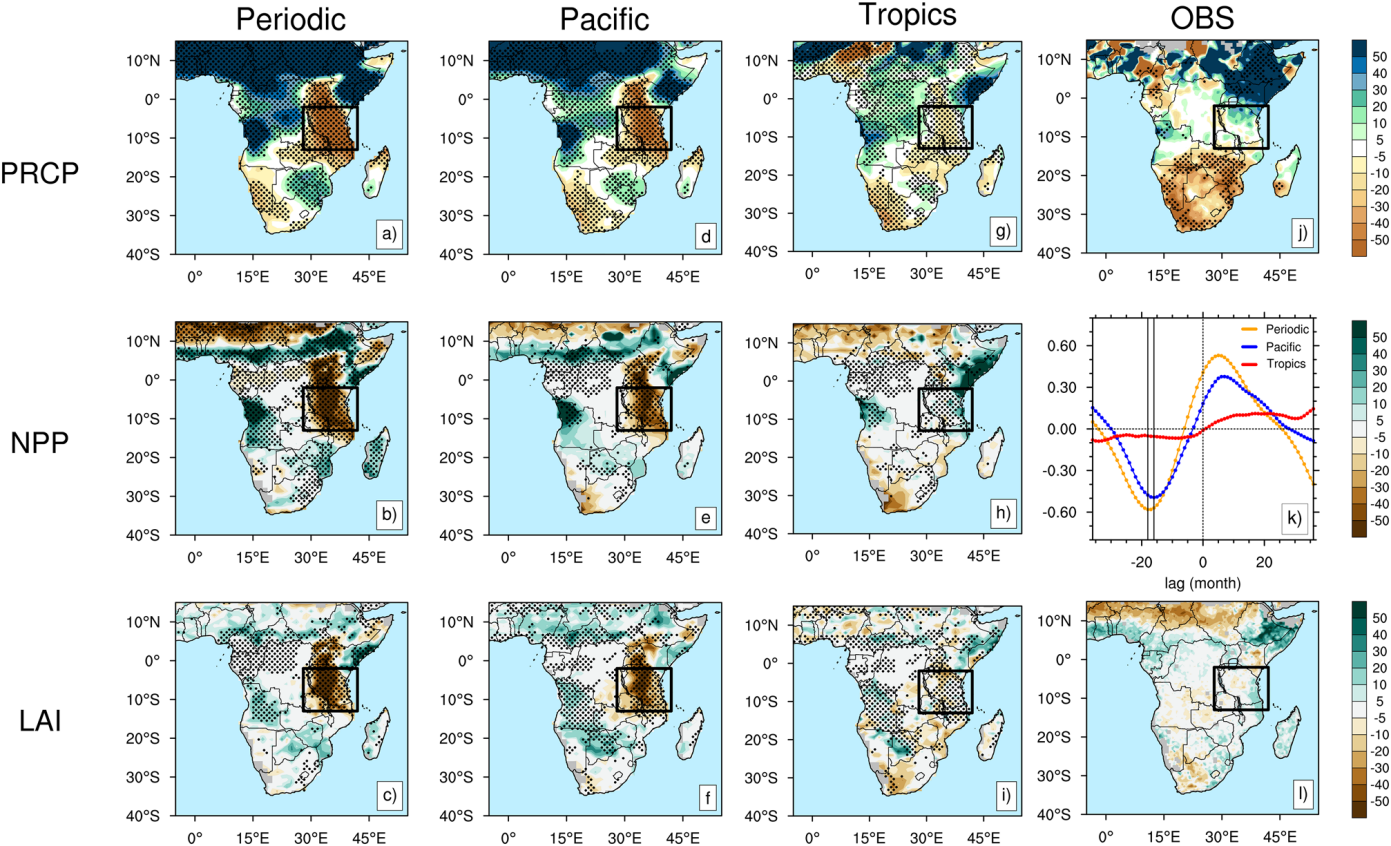
Composite differences (unit: %) between El Niño and La Niña events for precipitation (PRCP) anomalies in D(0)JF(1), net primary production (NPP) anomalies in JFM(1), and Leaf Area Index (LAI) anomalies in MJJ(2) for the Periodic experiment (**a**–**c**), the Pacific experiment (**d**–**f**), the Tropics experiment (**g**–**i**), as well as PRCP and LAI for the observations (**j**, **l**). Stippling indicates a statistically significant difference at the 95% significance level. Black box shows the surrounding Tanzania region (2–13°S, 28–42°E). The lead-lag cross-correlation between the Niño3.4 index and LAI anomalies over Tanzania for the Periodic experiment (yellow), the Pacific experiment (blue), and the Tropics experiment (red) (**k**). Fig. 2 was generated using NCAR Command Language Version 6.5.0 (http://dx.doi.org/10.5065/D6WD3XH5).

To understand first the symmetric (i.e., linear) response to El Niño and La Niña events, we show El Niño minus La Niña composites. Observed composite differences between El Niño and La Niña events display pronounced positive precipitation anomalies over the Horn of Africa and negative anomalies over Southern Africa in D(0)JF(1) (Fig. 2j). The three experiments (Periodic, Pacific, and Tropics) reproduce these anomalies reasonably well (Fig. 2a,d,g, Table S1).

Interestingly, the Periodic and the Pacific experiments exhibit a 50 % D(0)JF(1) rainfall reduction over Tanzania for the El Niño minus La Niña composite (Fig. 2a,d) and an accompanying negative Net Primary Production (NPP) anomaly during January–February–March of the decaying ENSO year [JFM(1)] (Fig. 2b,e). Mostly, the stronger NPP anomalies prevail over grassland and deciduous forest (herbaceous cover) along with a larger precipitation response, in accordance with previous studies^2^. In contrast, the observations and the more realistic Tropics experiment show only very weak rainfall, NPP, and Leaf Area Index (LAI) anomalies over Tanzania (Fig. 2g,h,i,j,l). Agreeing with other studies^29^, this finding suggests that tropical Indian or Atlantic Ocean SST anomalies might play an important role in muting the direct Pacific response over this region. We hypothesize specifically that the negligible observed rainfall response over Tanzania in the observations can be attributed to compensating effects from the direct Pacific effect and the El Niño-related Indian Ocean warming effect on the Walker circulation (Fig. 1b,c).

This hypothesis is further supported by the lead-lag cross-correlation relationship between ENSO and LAI anomalies in the Periodic and the Pacific experiments (Fig. 2k). According to this analysis, ENSO is leading LAI anomalies in Tanzania by about one year in these two experiments, whereas no statistically significant correlation can be found in the Tropics experiment. ENSO negatively correlates with LAI over Tanzania at a maximum lag of 16-months (*R* = 0.49, *p* < 0.00001) in the Pacific and 18-months (*R* = 0.58, *p* < 0.00001) in the Periodic experiments (Fig. 2k). In contrast, for the Tropics experiment, the correlation is not significant (*R* = 0.06, *p* = 0.11) (Fig. 2k). Regarding the LAI response to ENSO at this 16-18 months lag (that is, in May-June-July in year 2 after the ENSO event peak time: MJJ(2)), we find larger negative anomalies over Tanzania in the Pacific and the Periodic experiments (Fig. 2c,f), while they are much weaker anomalies in the Tropics experiment and the observations (Fig. 2i,l).

To further support our hypothesis, we compare CLM4 NPP and LAI changes to those obtained with the offline vegetation model BIOME4 which has been forced by the CESM1.2.2 monthly output of the sensitivity experiments (see Data and Experiments). The simulated annual mean NPP response over Africa to ENSO represented by the CLM4 agrees well with BIOME4 (Fig. S1-2). However, negative anomalies of annual mean LAI over Tanzania persist in CLM4 for much longer than in the BIOME4 experiments (Fig. S3-4), which is related to the fact that the BIOME4 model does not integrate the climate forcings of the previous months, but finds an equilibrium solution for each monthly forcing, in contrast to the CLM4.

**Figure. 3:**
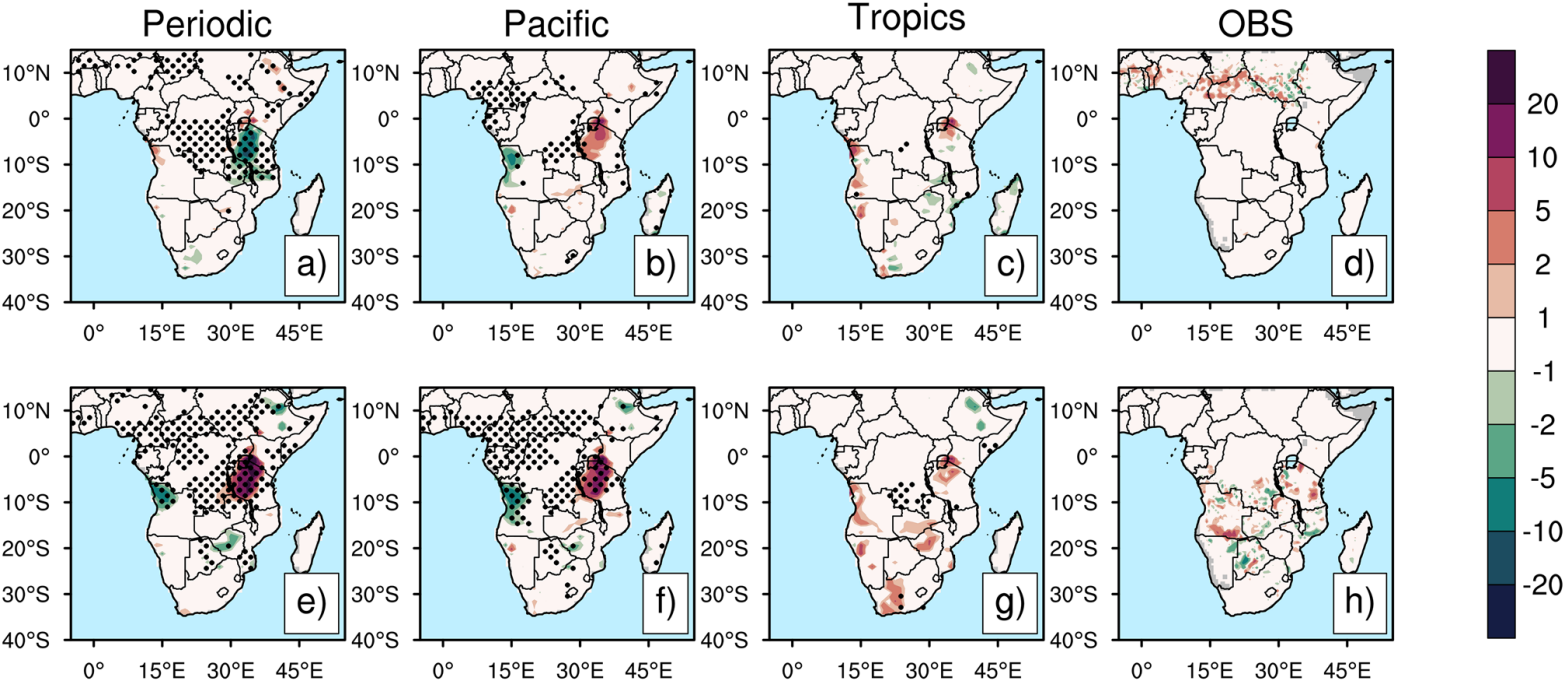
Composite differences of burned area (unit: %) between El Niño and La Niña events for the Periodic experiment, the Pacific experiment, the Tropics experiment, and the observations for D(0)JF(1) (**a**–**d**) and SON(1) (**e**–**h**): El Niño events: 1997, 2002, 2009; La Niña events: 1999, 2000, 2007, 2010 for observations. Stippling indicates a statistically significant difference at the 95% significance level. Fig. 3 was generated using NCAR Command Language Version 6.5.0 (http://dx.doi.org/10.5065/D6WD3XH5).

Moreover, over Tanzania, the delayed response to ENSO is also found in the CLM4 in wildfire activity (Fig. 3). The periodic experiment shows negative anomalies in burned area over Tanzania in D(0)JF(1) and statistically insignificant differences in the Pacific and Tropics experiments, as well as in the observations. However, the Periodic and the Pacific experiments show a 10–20 % increase in burned area over Tanzania in September-October-November in year 1 after ENSO event peak time [SON(1)] (Fig. 3a-d), whereas the observations and the Tropics experiment show statistically insignificant differences (Fig. 3e-h).

### Combination mode-driven rainfall response over Tanzania

**Figure. 4:**
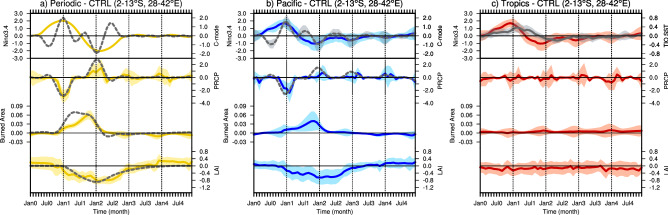
Time evolution over Tanzania (2–13°S, 28–42°E) for the Periodic experiment (**a**), the Pacific experiment (**b**) and the Tropics experiment (**c**): Niño3.4 index (solid yellow/blue/red line; unit: K), C-mode index (dashed gray line; unit: K), Tropical Indian Ocean (TIO) SST anomalies (solid gray line; unit: K), precipitation anomalies (solid yellow/blue/red line; unit: mm/day), reconstructed precipitation anomalies (dashed gray line), burned area (solid yellow/blue/red line; unit: fraction), reconstructed burned area anomalies (dashed gray line), LAI anomalies (solid yellow/blue/red line; unit: m^2^/m^2^), and reconstructed LAI anomalies (dashed gray line). Transparent shading indicates ± 1 standard deviation. Fig. 4 was generated using NCAR Command Language Version 6.5.0 (http://dx.doi.org/10.5065/D6WD3XH5).

The temporal evolution of the rainfall response over Tanzania to ENSO shows a rapid transition during the peak phase of both El Niño and La Niña in both the Periodic and the Pacific experiments, but not in the Tropics experiment (Fig. 4). The rainfall response is particularly pronounced in the former during the peak phase of ENSO in D(0)JF(1), which is also the climatological wet season (Fig. 4, Fig. S5). This illustrates the tight coupling between climatological conditions and the imposed ENSO signal. To further understand the distinct atmospheric response to ENSO in the absence of TIO SST anomalies, we hypothesize that the precipitation response over Tanzania to ENSO is driven by the seasonally modulated interannual ENSO variability, which is referred to as a Combination mode (C-mode)^30^. According to this simple model, the precipitation anomalies can be written as

1 $$P^{*} \left( t \right) = {\upalpha }{\text{ENSO}}\left( t \right) + ~{\upbeta }{\text{ENSO}}\left( t \right) \cdot \cos \left( {\omega _{a} t} \right),$$

where $\upalpha \;{\text{and}}\;\upbeta$ are the regression coefficients for the ENSO and theoretical C-mode predictors, and $\omega_{a}$ the frequency of the annual cycle. The phase of the annual cycle has a minimum value in July and a maximum in January. ENSO variability here is characterized by the Niño3.4 SST anomalies. One can also include a white noise precipitation forcing, but since we consider ensemble mean properties in a linear model, the noise forcing is not necessary to understand the temporal evolution. The time-series in the Periodic and Pacific experiments show that the reconstructions of precipitation anomalies over Tanzania via the C-mode equation reproduce the seasonally varying simulated rainfall response to ENSO well (Periodic: R = 0.64, *p* < 0.00001; Pacific: R = 0.65, *p* < 0.00001) (Fig. 4). The simulated La Niña response is somewhat reduced as compared to the El Niño rainfall anomaly. This is reminiscent of an atmospheric nonlinearity to otherwise symmetric SST forcing.

### Role of wildfires in the vegetation response to ENSO

In the absence of TIO warming in the Periodic and Pacific experiments, El Niño-induced drying increases the occurrence of fires, which is manifest in the prolonged positive anomalies in burned area lasting for about one year after the peak of El Niño (Fig. 4a,b). In CLM4, the probability of fire occurrence is parameterized by fuel availability and near-surface soil moisture conditions^31^. The annual burned area is driven by fire seasonal length and fire resistance depends on plant functional type (PFT)^32,33^. In addition, the water balance for soil moisture is controlled by precipitation, evaporation, and runoff, etc., but precipitation is the main driver for eastern Africa. To investigate the dynamical linkage between precipitation and wildfire responses to ENSO, we hypothesize that changes in burned area *B*, are driven by precipitation variability P^*^. Here we choose P^*^ as the ENSO-reconstructed precipitation anomaly from Eq. (1). We assume in its simplest linearized form that the burned area does not depend on the available vegetation, which allows us to introduce a fixed mean recovery timescale (${{\mu }_{1}}^{-1}$), in which the burned area can regrow. The simplified linearized model then reads:

2 $$\frac{{dB\left( t \right)}}{{dt}} = - ~\mu _{1} B(t) - \theta _{1} P^{*}\left( t \right).$$

Appropriate parameters values derived from the CESM1.2.2 and CLM4 output are given in Table S2. In the Periodic experiment, the reconstruction of burned area response over Tanzania captures the simulated temporal evolution reasonably well (R = 0.82, *p* < 0.00001), suggesting that the burned area response can be determined essentially by the time integral of the direct ENSO effect and the C-mode term. Previous studies support the notion that the lagged response of wildfire activity in some areas can be linked to the integrated effect of antecedent precipitation anomalies^7,34^. In the Periodic experiment, less rainfall over Tanzania during the wet season [D(0)JF(1)] and successive dry season promote a lagged response in burned area in SON(1) (Fig. S5). Subsequently, LAI anomalies over Tanzania slowly develop after the peak of El Niño and are prolonged until the following La Niña event. Especially, the peak of negative anomalies in LAI occurs during the mature La Niña phase in December-January–February in year 2 [DJF(2)], in spite of the maximum rainfall anomalies during this time (Fig. 4, Fig. S5). The vegetation response to climate factors also depends on vegetation resistance and resilience^35^. The vegetation carbon pool in CLM4 is governed by new growth, litterfall, mortality, and fire processes. The resilience of vegetation response can be represented by the process of new growth of post-fire vegetation. Accordingly, we hypothesize that the LAI response can be largely explained by the integrated effect of burned area (from Eq. 2), where *L* represents temporal variation of LAI, and ${\mu }_{2}$ is about 8 month^−1^ as an inverse damping time scale (characterizing vegetation resilience):

3 $$\frac{{dL\left( t \right)}}{{dt}} = - ~\mu _{2} L(t) - \theta _{2} B\left( t \right).$$

The parameters values in equation 3 were calculated by optimizing the first-order autoregressive (AR(1)) model (Tables S2, S3). According to this simplified double-integration model (Eqs. 1–3), the LAI response over Tanzania correlates highly with simulated LAI anomalies in the Periodic forcing experiment (R = 0.72, *p* < 0.00001), indicating that the lagged and prolonged vegetation response to ENSO is explained by vegetation resilience and the integrated effect of wildfire activity. Similar double-integration models have been introduced to explain also the emergence of low-frequency marine biogeochemical variability^36^.

## Discussion and conclusions

In this study, we explored how vegetation in the southeastern part of Africa changes in response to interannual ENSO variability through a series of model experiments. Focusing on Tanzania, we found that, in the absence of TIO variability, the rapid transition of precipitation anomalies during ENSO events is determined by the interaction between ENSO and the annual cycle of rainfall over Tanzania (the so-called C-mode). After the occurrence of El Niño, the pronounced decrease in rainfall over Tanzania leads to an enhancement in burned area with a time delay, thereby prolonging a marked vegetation decrease for 2 years. This response can be explained by the integrated effect of wildfire (double integrated effect of precipitation) and vegetation resilience through an idealized dynamical model, which explains the AGCM results reasonably well. In contrast, the BIOME4 offline experiment forced with the data from the CESM1.2.2 sensitivity experiment does not capture this effect, (Figs. S2, S4), because it calculates the equilibrium for each monthly forcing and does not account for the time history.

However, for the Tropics experiment (as well as in the observations) we do not find strong ENSO-related anomalies in precipitation, wildfire, and vegetation over Tanzania. This is because TIO warming during El Niño events compensates the rainfall response to ENSO over Tanzania (Fig. 4c) by weakening the anomalous atmospheric subsidence (Fig. 1b,c). This offset response is consistent with the opposite impact between Indian Ocean Basin-wide mode (IOBM) and ENSO on seasonal rainfall variability over Africa discussed in previous studies^37^. The IOD is another primary climate factor, which can affect rainfall and vegetation variability over eastern Africa^2,37–39^, but the IOD impact to eastern Africa peaks in September–November [SON(0)] (Fig. S6). This is too early to cause major precipitation and vegetation anomalies in Tanzania (Fig. 4, Fig. S6).

A caveat to be mentioned here is that the current land carbon models have limitations in realistically simulating climate-fire-vegetation interactions. In particular, the CLM4 in our model-set-up does not consider the human impact of wildfire activity (e.g., ignition, suppression, etc.), which implies that the model could overestimate the wildfire response to climate conditions. Other complications may arise from the fact that the interannual response of vegetation to climate variability in the observations may further depend on the vegetation structure^2^ and spatial fragmentation, which are not considered in CLM4.

Despite the modeling caveats, our study provides new conceptual insights into the physical mechanisms of climate-fire-vegetation interactions over eastern Africa. The double-integration model (Eqs. 1–3) proves to be useful in understanding time-variability and leads and lags between climate forcing, fire, and vegetation responses. It can also be applied to understand the effect of projected future changes in ENSO and TIO^40–42^ on fire and vegetation in eastern Africa.

## Data and experiments

### Observations

We used precipitation data from Global Precipitation Analysis Products of the Global Precipitation Climatology Centre (GPCC)^43^ and University of East Anglia’s Climate Research Unit (CRU)^44^, 200 hPa wind from European Centre for Medium-Range Weather Forecasts (ECMWF) reanalysis generation 5 (ERA5)^45^, and SST from the Hadley Centre Sea Ice and Sea Surface Temperature data set version 1 (HadISST1)^46^. To characterize observed vegetation changes, we utilized leaf area index (LAI) data derived from the Global Inventory Modeling and Mapping Studies (GIMMS) Normalized Difference Vegetation Index (NDVI3g) for the period 1982 to 2011^47^. The monthly Global Fire Emissions Database version 4 (GFEDv4)^48^ was used to characterize the 1994–2014 wildfire activity.

### Model and experiments

We conducted a suite of atmospheric general circulation model (AGCM) experiments with the Community Earth System Model (CESM 1.2.2) using the Community Atmosphere Model version 4.0 (CAM4)^49^ coupled to the Community Land Model version 4.0 (CLM4)^33,50^ with active Carbon-Nitrogen (CN) biogeochemistry (https://svn-ccsm-models.cgd.ucar.edu/cesm1/release_tags/cesm1_2_2/).

The model, which uses a horizontal resolution of approximately 1-degree, was spun up until the carbon and nitrogen pools were equilibrated to a 1957-2016 SST climatology boundary forcing and present-day greenhouse gas concentrations. We then performed four different types of AGCM model experiment ensembles to investigate the vegetation response over sub-Saharan Africa to interannual tropical SST variability starting from these equilibrated initial conditions. The ensemble simulations were performed with different initial conditions. These were generated by applying small perturbations of round-off error magnitude to the initial temperature field using the CESM namelist variable “pertlim”^51^.

First, a control experiment (CTRL) was carried out with a repeating global climatological SST forcing for the period 1957–2016 using a 3-member ensemble. The CTRL roughly reproduces the observed annual precipitation (PRCP) and LAI climatological patterns over Africa (Fig. S7, Table S5). The simulated seasonal mean precipitation over Africa region against observations are comparable to CMIP5 models^52^ (Table S4). The CTRL simulation poorly simulates annual burned area over sub-Saharan Africa, but captures the spatial pattern of the annual burned area over eastern Africa reasonably well (Table S5).

To illustrate the impact of observed ENSO variability, a “Pacific” experiment was conducted by adding the observed SST anomalies over the tropical eastern Pacific (15°S–15°N, 180°-90°W) for the period 1957–2016 to the climatology with a 10-member ensemble. A “Tropics” experiment was forced with SST anomalies over the whole tropics (15°S–15°N) for the period 1957–2016 to investigate the response to other modes of pantropical SST variability in addition to ENSO with a 3-member ensemble. An idealized “Periodic” experiment was designed to investigate the response to symmetric ENSO variability (see for instance Stuecker et al*.*^53^). The regressed ENSO SST anomaly pattern over the tropical eastern Pacific with an idealized sinusoidal 2.5 years periodicity was added to the observed SST climatology (1957–2016) and the experiment was run for 100 years with a 3-member ensemble. The climate response in all perturbation experiments is defined relative to the control experiment climate. Outside the tropical SST perturbation regions, the SST is the same as in the CTRL simulation.

To further determine the robustness of the vegetation response to tropical SST-driven climate, we performed offline simulations with dynamic and equilibrium global vegetation model, BIOME4, at a 0.5-degree resolution^54^, which can simulate biogeography of vegetation. BIOME4 was forced under present-day atmospheric CO_2_ concentrations (367 ppm) using monthly climatological values of minimum surface temperature, surface temperature, cloudiness, and precipitation obtained from the CESM1.2.2 “Pacific”, “Tropics”, and “Periodic” experiments (see supplementary figures S2, S4).

## Supplementary Information

Supplementary Information.
