# Supplementary material for: Tropical Indo-Pacific SST influences on vegetation variability in eastern Africa
Source: Sci Rep. 2021 May 17;11:10462. doi: 10.1038/s41598-021-89824-x (PMC8129105; doi:10.1038/s41598-021-89824-x)
Supplement: Supplementary file 1 — Supplementary Information. [file 41598_2021_89824_MOESM1_ESM.docx]

**Supplementary Material**

Tropical Indo-Pacific SST influences on vegetation variability in eastern Africa

In-Won Kim*, Malte F. Stuecker, Axel Timmermann, Elke Zeller, Jong-Seong Kug, So-Won Park, and Jin-Soo Kim

*corresponding author: iwkimi@pusan.ac.kr


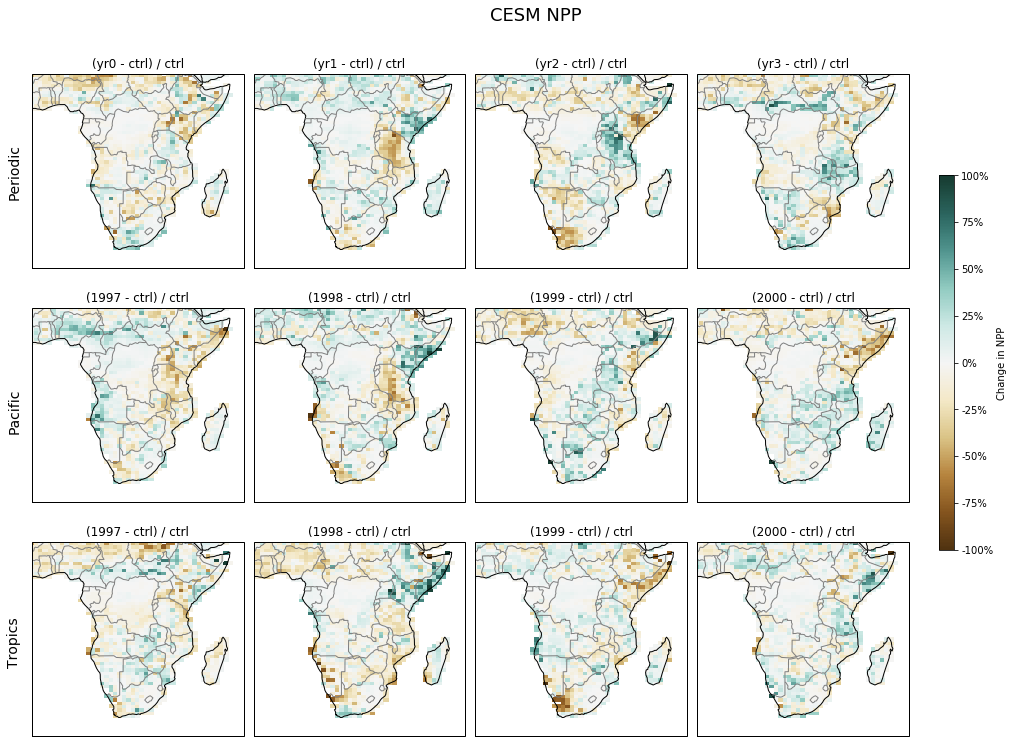


Figure S1. Annual-mean NPP anomalies (unit: %) for the Periodic experiment, the Pacific experiment, and the Tropics experiment compared to the CTRL as simulated by CLM4. Time period for the Pacific and the Tropics experiments: 1997-2010 and years 1-4 for the Periodic experiment (ENSO transition phase from El Niño to La Niña). Figure S1 was generated using Python (https://www.python.org/).


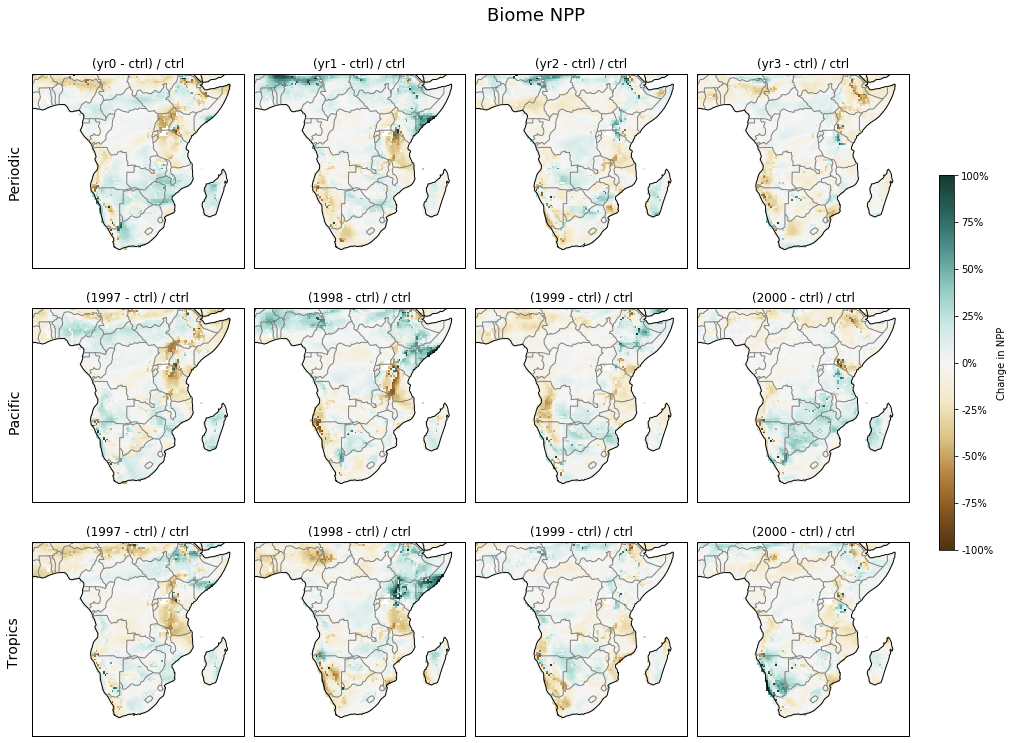


Figure S2. Annual-mean NPP anomalies (unit: %) for the Periodic experiment, the Pacific experiment, and the Tropics experiment compared to the CTRL as simulated by BIOME4. Time period for the Pacific and the Tropics experiments: 1997-2010 and years 1-4 for the Periodic experiment (ENSO transition phase from El Niño to La Niña). Figure S2 was generated using Python (https://www.python.org/).


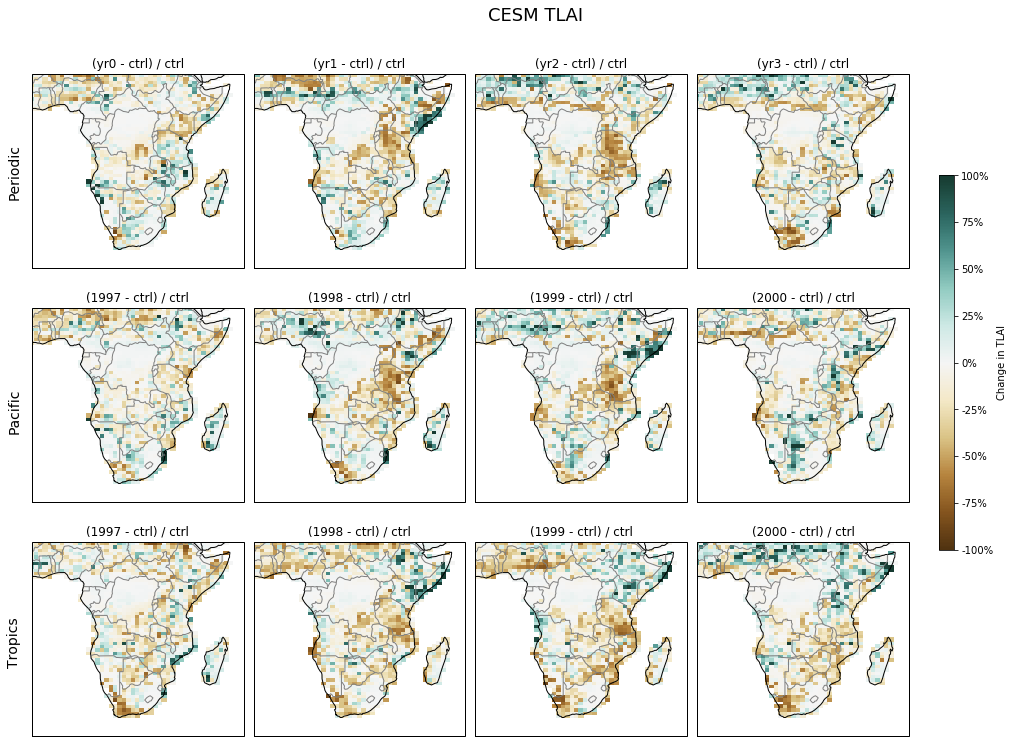


Figure S3. Annual-mean LAI anomalies (unit: %) for the Periodic experiment, the Pacific experiment, and the Tropics experiment compared to the CTRL as simulated by CLM4. Time period for the Pacific and the Tropics experiments: 1997-2010 and years 1-4 for the Periodic experiment (ENSO transition phase from El Niño to La Niña). Figure S3 was generated using Python (https://www.python.org/).


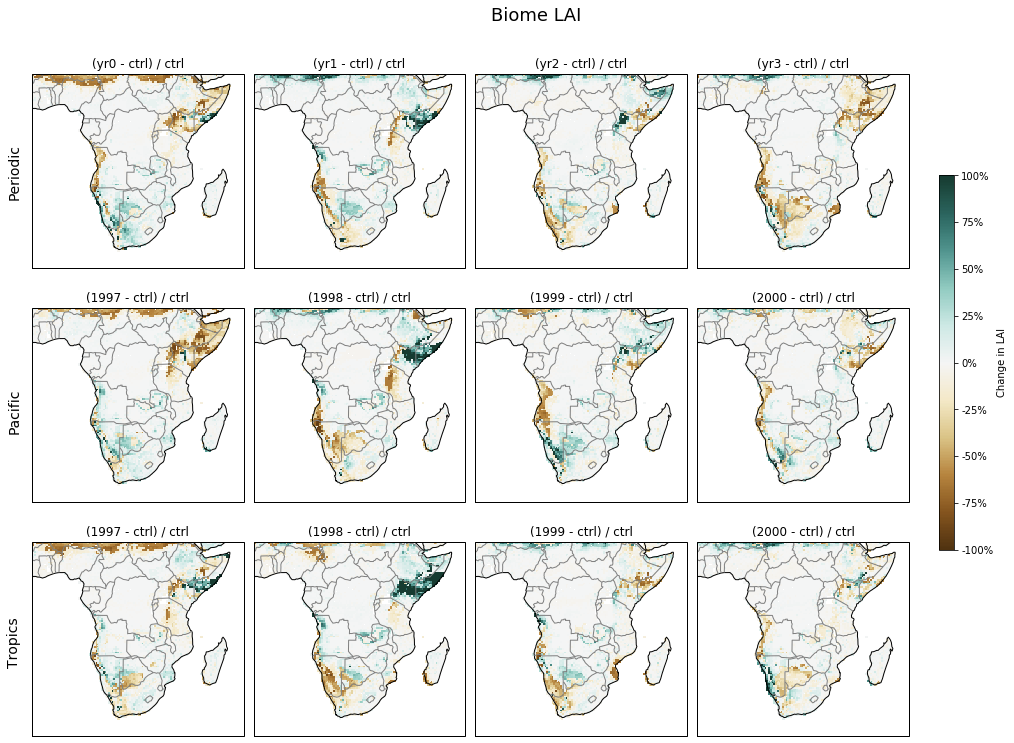


Figure S4. Annual-mean LAI anomalies (unit: %) for the Periodic experiment, the Pacific experiment, and the Tropics experiment compared to the CTRL as simulated by BIOME4. Time period for the Pacific and the Tropics experiments: 1997-2010 and years 1-4 for the Periodic experiment (ENSO transition phase from El Niño to La Niña). Figure S4 was generated using Python (https://www.python.org/).

Figure S5. Monthly evolution over Tanzania (2-13°S, 28-42°E) for the Periodic experiment: Niño3.4 index (solid yellow line; unit: K), precipitation (unit: mm/day), upper soil moisture (mm3/mm3), fire seasonal length (day), burned area (unit: fraction), Photosynthesis (unit: mol/m^2^day), and LAI (unit: m^2^/m^2^). Figure S5 was generated using NCAR Command Language Version 6.5.0 (http://dx.doi.org/10.5065/D6WD3XH5).

Figure S6. Time evolution over Tanzania (2-13°S, 28-42°E) for the Periodic experiment (b), the Pacific experiment (b), and the Tropics experiment (c): Niño3.4 index (solid red line; unit: K), Dipole Mode Index (DMI; solid gray line; unit: K), precipitation anomalies (unit: mm/day), burned area anomalies (unit: fraction), and LAI anomalies (unit: m2/m2). Transparent shading indicates ± 1 standard deviation. Figure S6 was generated using NCAR Command Language Version 6.5.0 (http://dx.doi.org/10.5065/D6WD3XH5).

Figure S7. Climatology of PRCP (unit: mm/day), LAI (unit: m2/m2), and burned area (unit: fraction) over Africa in observations (upper, a-c) and the control run (lower, d-f). Figure S1 was generated using NCAR Command Language Version 6.5.0 (http://dx.doi.org/10.5065/D6WD3XH5).

| Experiments | Periodic | Pacific | Tropics |
| --- | --- | --- | --- |
| Horn of Africa | 0.56 | 0.41 | 0.65 |
| Tanzania | 0.31 | 0.16 | 0.53 |
| Southern Africa | 0.69 | 0.86 | 0.87 |

**Table S1.** Pattern correlation coefficients (PCC) between three experiments and observation in composite differences of precipitation anomalies in D(0)JF(1) between El Niño and La Niña events

**Table S2.** Parameters for equations (1)-(3)

| Parameter | Description | Value | Unit |
| --- | --- | --- | --- |
| $\alpha$ | Regression coefficient of precipitation anomaly on ENSO | -0.36 | K |
| $\beta$ | Regression coefficient of precipitation anomaly on C-mode | -0.61 | K |
| $\mu_{1}$ | Recovery timescale of burned area | 2.50 | fraction |
| $\theta_{1}$ | Burned area resistance against drought | 0.01 | mm/day |
| $\mu_{2}$ | Resilience timescale of vegetation | 8.33 | m^2^/m^2^ |
| $\theta_{2}$ | Vegetation resistance against burned area | 2.00 | fraction |

**Table S3.** Correlation coefficient (CC) and Mean Squared Error (MSE) between LAI anomalies and analytic solution with different values of $\boldsymbol{\mu}_{\boldsymbol{2}}$^-1^ (month^-1^) in the periodic experiments

| ${\boldsymbol{\mu}_{\boldsymbol{2}}}^{\boldsymbol{-1}}$ **(month^-1^)** | 0.5 | 0.4 | 0.3 | 0.2 | 0.15 | 0.13 | 0.12 | 0.1 | 0.08 |
| --- | --- | --- | --- | --- | --- | --- | --- | --- | --- |
| **CC** | 0.563 | 0.604 | 0.645 | 0.690 | 0.712 | 0.717 | 0.718 | 0.715 | 0.699 |
| **MSE** | 1.070 | 0.673 | 0.376 | 0.183 | 0.129 | 0.117 | 0.113 | 0.111 | 0.119 |

**Table S4.** Root mean square error (RMSE) and uncentered pattern correlation coefficients (PCC) between CRU, GPCC, and CTRL in seasonal mean precipitation over eastern Africa (11.5°S-15°N, 25°E-52°E), Western Africa (11.5°S-15°N, 20°W- 25°E), and Southern Africa (35-11.5°S,10°W-52°E)

| Region | Season | CRU | | GPCC | |
| --- | --- | --- | --- | --- | --- |
|  |  | RMSE | PCC | RMSE | PCC |
| Eastern Africa | MAM | 2.74 | 0.57 | 2.54 | 0.65 |
|  | JJA | 2.05 | 0.66 | 2.12 | 0.66 |
|  | SON | 2.99 | 0.82 | 2.77 | 0.82 |
|  | DJF | 2.87 | 0.89 | 2.64 | 0.90 |
| Western Africa | MAM | 2.39 | 0.80 | 2.39 | 0.90 |
|  | JJA | 1.67 | 0.80 | 2.01 | 0.78 |
|  | SON | 2.35 | 0.74 | 2.38 | 0.71 |
|  | DJF | 2.63 | 0.85 | 2.56 | 0.84 |
| Southern Africa | MAM | 1.22 | 0.76 | 1.22 | 0.78 |
|  | JJA | 0.48 | 0.78 | 0.55 | 0.80 |
|  | SON | 1.45 | 0.72 | 1.48 | 0.72 |
|  | DJF | 3.06 | 0.72 | 2.95 | 0.71 |

**Table S5.** Root mean square error (RMSE) and uncentered pattern correlation coefficients (PCC) between satellite-based estimates (GFEDv4, GIMMS) and CTRL in burned area and LAI over eastern Africa (11.5°S-15°N, 25°E-52°E), Western Africa (11.5°S-15°N, 20°W- 25°E), and Southern Africa (35-11.5°S,10°W-52°E)

| Region | Burned area | | LAI | |
| --- | --- | --- | --- | --- |
|  | RMSE | PCC | RMSE | PCC |
| Eastern Africa | 5.10 | 0.35 | 3.40 | 0.75 |
| Western Africa | 6.55 | -0.13 | 4.27 | 0.81 |
| Southern Africa | 3.57 | 0.06 | 2.61 | 0.45 |
